# Therapeutic drug monitoring and the conservative management of chronic tuberculous empyema: case report and review of the literature

**DOI:** 10.1186/s12879-015-1093-7

**Published:** 2015-08-12

**Authors:** Richard Long, James Barrie, Charles A. Peloquin

**Affiliations:** Department of Medicine, University of Alberta, Edmonton, Canada; Department of Radiology, University of Alberta, Edmonton, Canada; The Infectious Disease Pharmacokinetics Laboratory, Gainesville, Florida USA; College of Pharmacy and Emerging Pathogens Institute, University of Florida, 1600 SW Archer Rd; Rm P4-33, P.O. Box 100486, Gainesville, 32610-0486 Florida USA

**Keywords:** Chronic tuberculous emypema, Therapeutic drug monitoring

## Abstract

**Background:**

Chronic tuberculous empyema (CTE) is a rare and unusual, low grade and protracted, infection of the pleural space resulting in marked thickening, even calcification of the visceral and parietal pleura. Historically its management has been extraordinarily challenging. Differential penetration of anti-TB drugs into the pleural space has resulted in acquired drug resistance and surgery to remove the empyema or close a complicating bronchopleural fistula (BPF) has been technically difficult or unacceptably hazardous. On the basis of limited experience, the combination of tube thoracostomy or catheter drainage and high-end dosing of anti-TB drugs has been recommended as an initial approach to these lesions. Herein we report the first well documented case of closure of a BPF and cure of a CTE using this approach. The chances of a favorable outcome are improved, we suggest, by using therapeutic drug monitoring (TDM) to guide high-end drug dosing.

**Case Presentation:**

An 84 year old male immigrant to Canada from Croatia was diagnosed with a CTE after he developed a BPF. The diagnosis was made 62 years after what was, in retrospect, an episode of tuberculous pleurisy. He was treated with computed tomography-guided catheter drainage and TDM-guided high-end dosed anti-TB drugs (serum and pleural fluid drug concentrations) over a 10 month period. Sustained closure of the BPF and mycobacteriologic cure of the CTE was achieved. Drug concentrations in the present case and all other reported cases are summarized and interpreted.

**Conclusion:**

When serum concentrations of the anti-TB drugs isoniazid, pyrazinamide and ethambutol at the high end of the normal range are achieved, pleural fluid concentrations at the low end of the normal range may be anticipated in CTE. Though highly protein bound drugs such as rifampin and moxifloxacin appear to penetrate CTEs less well, their free concentrations in the pleural space may be proportionately higher on account of lower protein concentrations. Interventional radiology and TDM increase the chances that conservative management of CTE will be successful.

## Background

Tuberculous empyema is a chronic active infection of the pleural space resulting in marked thickening, even calcification, of the visceral and parietal pleura [1–3]. With few exceptions it is a late complication of a primary tuberculous pleural effusion or more rarely now that effective anti-tuberculosis (TB) drugs are available, collapse therapy (artificial pneumothorax, thoracoplasty etc.) for cavitary pulmonary TB [1–3]. With respect to its pathogenesis as a complication of primary tuberculous effusion, it is perhaps best considered in the context of the latter’s natural history: weeks after primary infection a subpleural caseous focus ruptures into the pleural space where mycobacterial antigens interact with previously sensitized T cells to produce a delayed type hypersensitivity reaction, altered permeability and impaired pleural space clearing [2, 3]. The resulting tuberculous pleural effusion is usually self-limited, resolving over 4–16 weeks whether or not anti-TB drugs are given, though some residual pleural thickening may remain especially if the effusion is loculated [4, 5]. Untreated, such effusions carry a high risk of reactivation TB over the next 5 years [4]. Uncommonly, untreated effusions proceed directly to a tuberculous empyema. Within such empyemas the inflammatory process may be present for decades and, by virtue of being sequestered, be asymptomatic and discovered only after the patient undergoes a chest radiograph for another reason or develops a bronchopleural fistula (BPF) or *empyema necessitatis* [1–3].

Historically, the management of chronic tuberculous empyema (CTE) has been extraordinarily challenging. Differential penetration of anti-TB drugs through the thick fibro-calcific wall of the empyema has resulted in acquired drug resistance [6, 7], and surgery such as decortication to allow re-expansion of a trapped lung or decortication plus pneumonectomy to remove a lung that is predicted to cause ongoing morbidity is often technically difficult and/or unacceptably hazardous especially in an older patient.

Based on limited experience, tube thoracostomy along with high-end dosing of anti-TB drugs has been recommended as an initial approach to CTE [2, 3]. Evidence in support of this approach and a compelling rationale for its implementation follows.

## Case presentation

**Fig. 1:**
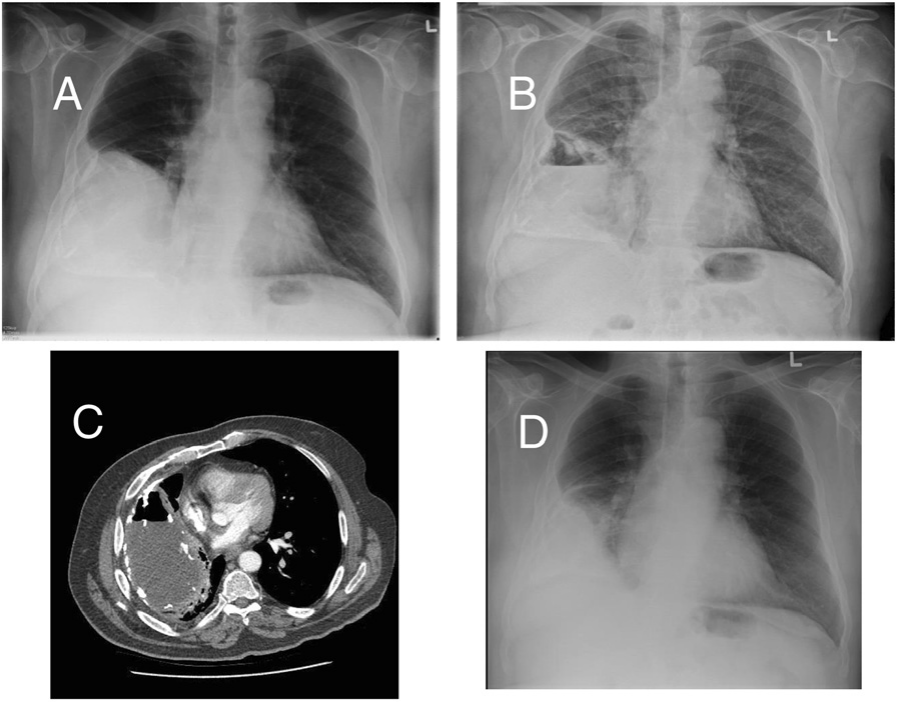
Panel **a**: A posterior-anterior (PA) chest radiograph dated November, 2010 showing a large loculated pleural effusion on the right side. Earlier radiographs (now purged) were reported to show a similar abnormality. Panel **b**: A PA chest radiograph dated January, 2012 showing an air-fluid level in the previously described loculated effusion. It is consistent with the interval development of a bronchpleural fistula. Panel **c**: A computed tomographic image dated June, 2012 showing right sided volume loss and a peripherally calcified loculated right hydropneumothorax. Panel **d**: A PA chest radiograph dated May, 2015 showing the previously described chronic loculated effusion to be reduced in size. An air-fluid level is no longer visible

An 84 year old male immigrant to Canada from Croatia was admitted to hospital in February, 2013 with a CTE. In 1950, while serving in the Croatian army, he recalled having chest pain and “water on the right lung”. He underwent percutaneous drainage of the “water” and the chest pain slowly resolved. Later, chest radiographs showed a large loculated right pleural effusion (Fig. 1a). All his adult life he worked as a carpenter without respiratory complaint. In retirement he was very active.

In November, 2011 he experienced the sudden onset of chest pain, shortness of breath, and productive cough. A chest radiograph revealed a new air fluid level at the right base consistent with a BPF (Fig. 1b). His cough was intermittently productive of tan colored phlegm and worse in the left lateral position.

Upon admission to hospital in February, 2013, co-morbidities included hypertension, hyperlipidemia and a clinical diagnosis of gout for which he was receiving allopurinol. HIV serology was negative. His weight was 82 kg. A computed tomographic (CT) scan of the thorax showed a large (14 × 10 × 10 cm) predominantly subpleural, peripherally calcified loculated right hydropneumothorax (Fig. 1c). Baseline renal and liver function was normal. A thoracentesis, performed under CT guidance, produced turbid tan colored fluid; total white blood count 154,000 × 10 [6]/L (mainly neutrophils), total protein 32 g/L (serum albumin and total protein 29 and 68 g/L, respectively), glucose <0.7 mmol/L (serum 4.2 mmol/L), lactic dehydrogenase >2700 U/L (serum 110 U/L), and pH 7.11. Cytology was negative. Sputum and pleural fluid cultures were positive for *Mycobacterium tuberculosis* susceptible to all first-line anti-TB drugs (indirect proportion method using BACTEC MGIT 960™, Becton, Dickinson and Company, Sparks, MD). Minimum inhibitory concentrations (MICs) were not measured. Pleural fluid aerobic, anaerobic and fungal cultures were negative. Age of the patient and existing recommendations, albeit limited in terms of their evidence base, suggested a conservative approach to management [2, 3]. A pleurX catheter was inserted under CT guidance and oral anti-TB drugs were started.

Anti-TB drug treatment consisted of 10 months of directly-observed isoniazid (INH), pyrazinamide (PZA), and ethambutol (EMB), 7.5 months of rifampin (RIF), and 3.5 months of a fluoroquinolone, either moxifloxacin (MOX) or levofloxacin (LEV). Although relatively contraindicated, given his history of gout, PZA was considered important to his regimen (see below), and fortunately was well tolerated. Simultaneous serum and pleural fluid anti-TB drug concentrations were measured after four weeks of daily treatment. Concentrations were measured at baseline, 1.5 (INH only), 2.5 and 6.5 hours after INH, RIF, PZA, EMB, and MOX in doses of 400, 600, 2000, 1400 and 600 mg, respectively. Thrice weekly INH, RIF, PZA, EMB, and LEV in doses of 900, 1200, 3000, 2000, and 1000 mg, respectively, were then introduced and after their introduction INH and RIF concentrations were again measured at 2, 6, and 8 hours post-dose. Drug concentrations were measured by the Infectious Disease Pharmacokinetics Laboratory in Gainesville, Florida, (http://idpl.pharmacy.ufl.edu/) using high performance liquid chromatography (HPLC)-ultraviolet for INH and RIF, gas chromatography and mass spectrometry for PZA and EMB and HPLC-florescence for MOX.

While maximum serum concentrations (C_max_) of all drugs were in the normal range (for RIF after twice the usual dose), there was delayed absorption of PZA, EMB and MOX; the time to C_max_ (T_max_) being at 6 hours rather than 2–3 hours (see Table 1). Pleural fluid to serum C_max_ ratios over the sampling intervals were low for all drugs (INH 0.50 after a 400 mg dose, 0.40 after a 900 mg dose; RIF 0.46 after a 600 mg dose, 0.12 after a 1200 mg dose; PZA 0.85 after a 2000 mg dose; EMB 0.48 after a 1400 mg dose; and MOX 0.28 after a 600 mg dose). With the exception of INH the concentrations of drugs in the pleural space were highest at the last sampling time point, suggesting that the actual C_max_ in the pleural space may not have been reached in the sampling interval. Daily and thrice weekly doses of drugs were adjusted upward as tolerated with the aim of achieving serum concentrations near the upper end of the normal range; INH 600 and 900–1200; RIF 900–1200; PZA 2000 and 3000; EMB 1400 and 2000–2400; and LEV 750–1000 mg, respectively. The smaller pill burden of daily dosing was better tolerated than larger pill burden of thrice weekly dosing. Otherwise treatment was uneventful.

**Table 1:** Reference Measures of In-vitro Activity and Pharmacokinetics and Reported C_max_ and T_max_ in Simultaneous Serum and Pleural Fluid Samples in Chronic Tuberculous Empyema

Reference Measures of In-Vitro Activity and Pharmacokinetics					Simultaneous Serum and Pleural C_max_ and T_max_ in CTE				
Drug	C_max_^a^	T_max_^a^	MIC^b^	C_max_/MIC	Dose (mg)	C_max_ (mg/L)		T_max_ (hrs)^c^	
	(mg/L)	(hrs)	(mg/L)		(Ref.)	Serum	Pleural Fluid	Serum	Pleural Fluid
Isoniazid	3-6	1-2	0.125	24.0-48.0	300 (12)	3.25	0.96	1 (6)	6 (6)
(300 mg)					400 (P1)	3.49	1.75	1 (6)	6 (6)
					900 (P2)	12.77	5.12	2 (8)	6 (8)
Rifampin	8-24	2	0.03125	256.0-768.0	600 (7)	21.75	0.87	2 (10)	10 (10)
(600 mg)					600 (12)	8.01	0.48	2 (6)	6 (6)
					600 (P1)	2.20	1.02	2 (6)	6 (6)
					1200 (P2)	12.92	1.57	2 (8)	8 (8)
Pyrazinamide	20-50	1-2	50.000	0.4-1.0	2000 (12)	45.82	22.68	1 (6)	6 (6)
(20–25 mg/kg)					2000 (P1)	34.99	29.85	6 (6)	6 (6)
Ethambutol	2-6	1-2	1.000	2.0-6.0	1200 (7)	1.60	1.90	6 (10)	6 (10)
(20–25 mg/kg)					1400 (P1)	3.31	1.60	6 (6)	6 (6)
Moxifloxacin	3-5	2	0.250	12.0-20.0	600 (P1)	3.80	1.08	6 (6)	6 (6)
(400 mg)					600 (7)^d^	10.08	4.84	2 (10)	10 (10)

Abbreviations: C_max_ maximum concentration; T_max_ time-to-C_max_ ; MIC minimum inhibitory concentration; CTE chronic tuberculous empyema; P1 present report, measurement after four weeks of treatment; P2 present report, measurement after six weeks of treatment.

^a^C_max_ and T_max_ measures were those in use by the Infectious Disease Pharmacokinetics Laboratory, Gainesville, Florida, USA. Free C_max_ concentrations are not shown, but on the basis of protein binding of 0 % for isoniazid, 85 % for rifampin, 10 % for pyrazinamide, 25 % for ethambutol and 50 % for moxifloxacin [8–10], are estimated to be 3.0-6.0, 1.2-3.6, 18–45, 1.5-4.5 and 1.5-2.5 mg/L, respectively

^b^MICs are those used by Gumbo et al. [11]

^c^Numbers in brackets refer to the sampling interval

^d^Ofloxacin

All sputum and pleural fluid specimens were negative for acid-fast bacilli on smear and culture after seven weeks of treatment, including specimens of both types collected immediately and one year following completion of treatment. The pleurX catheter was removed after two months of treatment; key drug concentration measurements had been made and the catheter site was uncomfortable when the patient lay in the right lateral decubitus position. The BPF closed spontaneously after six months of treatment (see Fig. 1d). At the time of writing, over 1.5 years post-completion of treatment, the BPF remained closed.

## Case context

Simultaneous serum and pleural fluid anti-TB drug concentrations have been measured in two other cases and these along with our own and reference measures are shown in the Table 1 [7–12]. For all drugs, pleural fluid C_max_ was lower and T_max_ was later (usually) than serum C_max_ and T_max_ over the sampling intervals. Clearly INH, PZA and EMB achieved better pleural fluid concentrations relative to serum than did RIF, though if we assume that the albumin fraction of the total protein concentration in the pleural fluid was similar to that in the serum, free RIF and MOX concentrations in our case may have been proportionately higher in the pleural space (in serum RIF and MOX are 85 % and 50 % protein bound, respectively) [8, 10]. Good penetration of INH is supported by the observation that INH resistance developed in all cases of CTE reported to have acquired drug resistance [2, 6, 7, 13]. In the two cases in which PZA was measured, it too appeared to penetrate well achieving pleural fluid concentrations that were 50-85 % of serum concentrations, a fortuitous event given that PZA works best in an acid environment and CTEs are characteristically acidic [2, 3, 14, 15]. In the two cases in which EMB was measured it too appeared to penetrate reasonably well achieving pleural fluid concentrations that were 50 % or more of serum concentrations, also fortuitous given that EMB is better at protecting INH against the acquisition of resistance than is PZA [16]. These data suggest that if serum INH, PZA and EMB concentrations close to the upper end of the normal range are achievable then pleural fluid concentrations close to the lower end of the normal range are achievable.

In the past it has been suggested that by dividing the measured pleural fluid C_max_ by the standard MICs of the drugs for susceptible isolates of *M. tuberculosis* (see Table 1), one obtain a C_max_/MIC ratio and that a ratio of greater than 4 be taken to indicate probable effectiveness [17]. Thus far this ratio has been of limited utility in CTE; [7, 12] after doses of INH (400 mg), RIF (1200 mg), PZA (2000 mg), EMB (1400 mg) and MOX (600 mg) in our own case, pleural fluid C_max_/MIC ratios were: 14.00, 50.24, 0.60, 1.60, and 4.20, respectively. However, it is to be noted that standard MICs and those used in the Table are based on expected ranges which have their own population confidence intervals [11]. Yet an individual isolate can have higher or lower MICs still within the “susceptible” range that will influence in vitro killing and would alter C_max_/MIC calculations. MICs were not measured in our case but might have been helpful. MIC testing should be considered in the management of future cases of such complexity.

## Conclusion

In this paper we provide a strong rationale for the conservative management of CTE, a lesion that despite its chronicity is remarkably well tolerated until it is complicated by either BPF or *empyema necessitatis*. When these complications occur the patient’s clinical status invariably deteriorates and, in the event of a BPF, they become a public health risk. Among well documented medical cures ours is the first to demonstrate closure of a BPF [2, 6, 12, 18]. Conservative management includes CT-guided catheter drainage and therapeutic drug monitoring (TDM)-guided anti-TB drugs.

In addition to minimizing the danger of lung contamination and possibly the acquisition of drug resistance [19], catheter drainage allows easy access to the pleural space for purposes of monitoring the mycobacteriologic response and measuring pleural fluid drug concentrations. Ideally catheters are positioned into the dorsal and caudal portion of the empyema under CT guidance [20].

Most importantly, conservative management includes TDM, allowing the clinician to make an informed decision about drug dosing, maximizing chances of cure and minimizing the risk of drug resistance. Dose–response relationships are known to exist for each of the anti-TB drugs used in standard therapy [21]. Lower doses and presumably lower serum concentrations are now considered suboptimal [21]. Although a minimum of two serum samples (2 and 6 hours post-dose) are normally recommended, in CTE peak pleural fluid concentrations of the anti-TB drugs are generally delayed and reduced relative to serum, suggesting the need for higher oral doses and a longer sampling interval. Lower pleural fluid drug concentrations may be the result of poor blood supply through the fibrosed calcified pleura, or of binding or inactivation of the drugs by material in the pleural space [7].

To locate the intrapleural concentrations of INH, PZA and EMB and possibly free RIF into the normal range it is necessary to target a serum concentration at the upper end of that range. Here the dose of drug that produces this target with an acceptable degree of toxicity is considered the maximum dose, not the “maximum” dose that may be listed in TB treatment guidelines [22]. If drug resistance or intolerance is present in patients whose case is uncomplicated by BPF, then combined oral and intrapleural drugs may be considered [12]. Once the irreducible pleural space is sterilized, and absent a BPF, it is reasonable to simply follow the case expectantly.

In summary, we believe the means are now at hand to recommend, with yet more confidence, a conservative approach to CTE. Because acute TB pleurisy may go undiagnosed, especially in resource poor settings, sporadic cases of CTE are likely to continue to occur.

### Consent

Written informed consent was obtained from the patient for publication of this case report and any accompanying images. A copy of the written consent is available for review by the editor of this journal.
